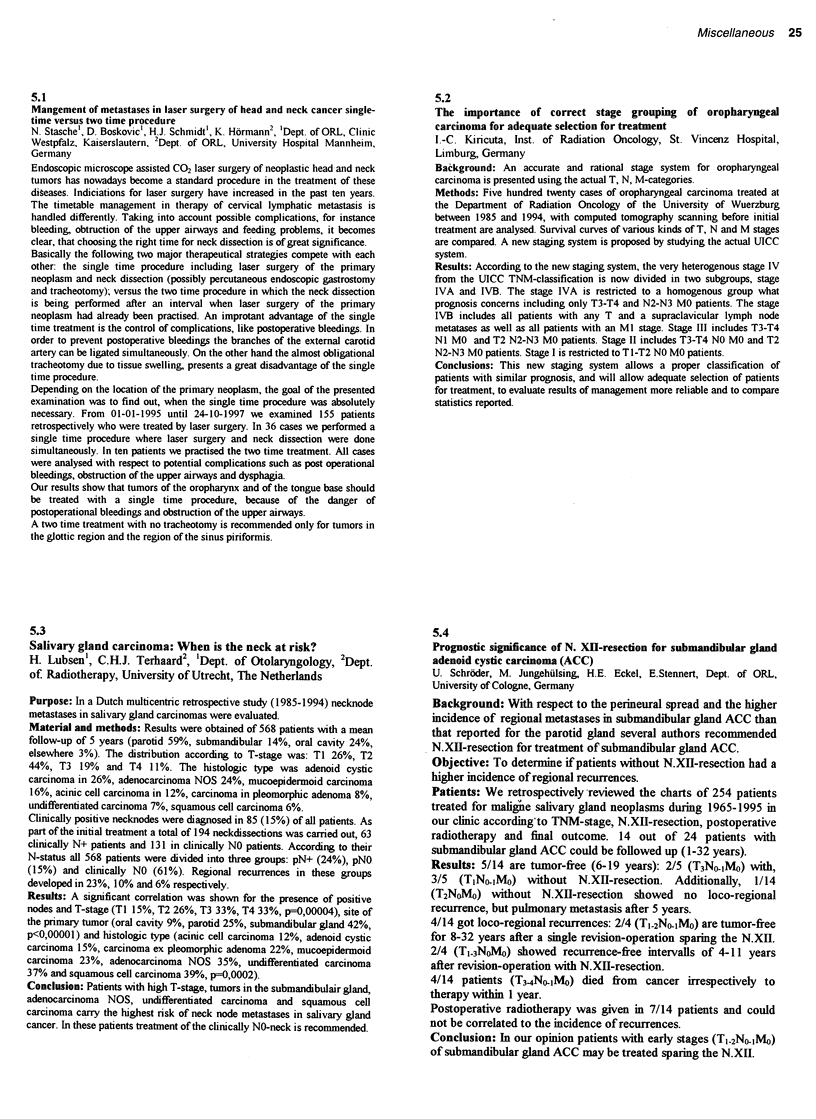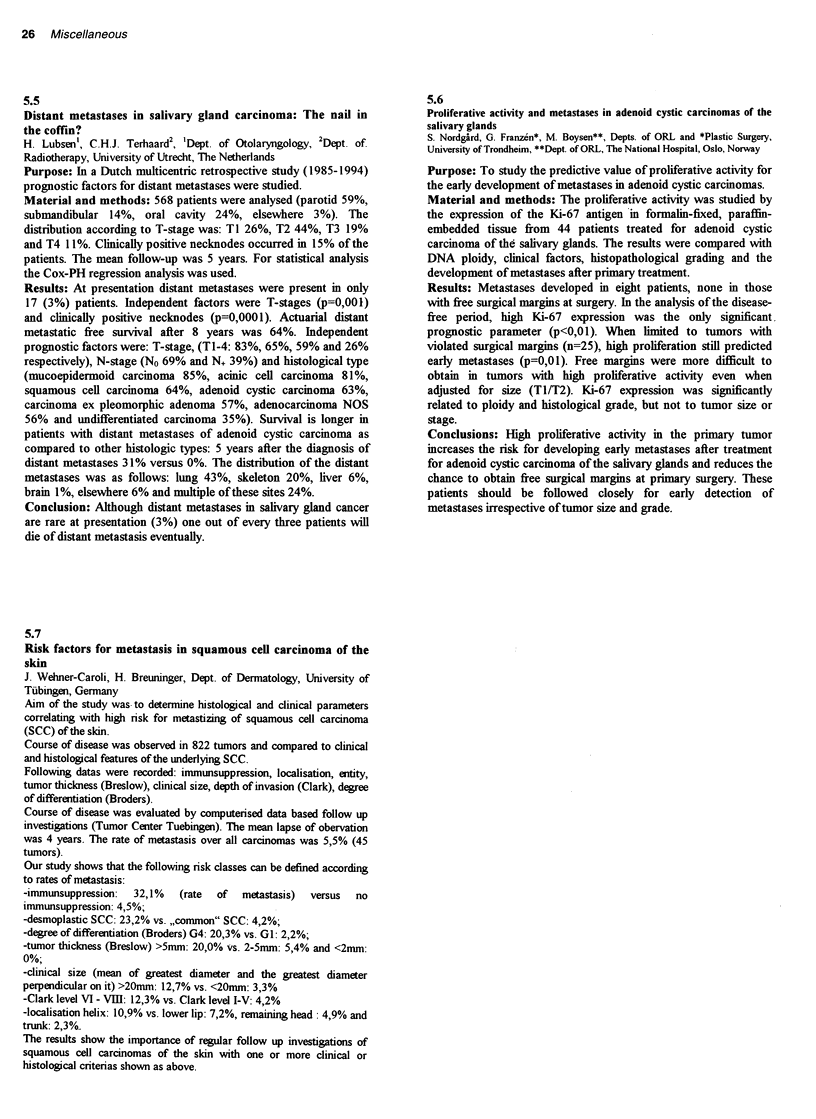# Miscellaneous

**Published:** 1998

**Authors:** 


**Mangement of metastases in laser surgery of head and neck cancer single-time versus two time procedure**

N. Stasche[1], D. Boskovic[1], H.J. Schmidt[1], K. Hörmann[2], [1]Dept. of ORL, Clinic Westpfalz, Kaiserslautern, [2]Dept. of ORL, University Hospital Mannheim, Germany

Endoscopic microscope assisted $CO_2$ laser surgery of neoplastic head and neck tumors has nowadays become a standard procedure in the treatment of these diseases. Indiciations for laser surgery have increased in the past ten years. The timetable management in therapy of cervical lymphatic metastasis is handled differently. Taking into account possible complications, for instance bleeding, obtruction of the upper airways and feeding problems, it becomes clear, that choosing the right time for neck dissection is of great significance.

Basically the following two major therapeutical strategies compete with each other: the single time procedure including laser surgery of the primary neoplasm and neck dissection (possibly percutaneous endoscopic gastrostomy and tracheotomy); versus the two time procedure in which the neck dissection is being performed after an interval when laser surgery of the primary neoplasm had already been practised. An improtant advantage of the single time treatment is the control of complications, like postoperative bleedings. In order to prevent postoperative bleedings the branches of the external carotid artery can be ligated simultaneously. On the other hand the almost obligational tracheotomy due to tissue swelling, presents a great disadvantage of the single time procedure.

Depending on the location of the primary neoplasm, the goal of the presented examination was to find out, when the single time procedure was absolutely necessary. From 01-01-1995 until 24-10-1997 we examined 155 patients retrospectively who were treated by laser surgery. In 36 cases we performed a single time procedure where laser surgery and neck dissection were done simultaneously. In ten patients we practised the two time treatment. All cases were analysed with respect to potential complications such as post operational bleedings, obstruction of the upper airways and dysphagia.

Our results show that tumors of the oropharynx and of the tongue base should be treated with a single time procedure, because of the danger of postoperational bleedings and obstruction of the upper airways.

A two time treatment with no tracheotomy is recommended only for tumors in the glottic region and the region of the sinus piriformis.

### 5.2

**The importance of correct stage grouping of oropharyngeal carcinoma for adequate selection for treatment**

I.-C. Kiricuta, Inst. of Radiation Oncology, St. Vincenz Hospital, Limburg, Germany

**Background:** An accurate and rational stage system for oropharyngeal carcinoma is presented using the actual T, N, M-categories.

**Methods:** Five hundred twenty cases of oropharyngeal carcinoma treated at the Department of Radiation Oncology of the University of Wuerzburg between 1985 and 1994, with computed tomography scanning before initial treatment are analysed. Survival curves of various kinds of T, N and M stages are compared. A new staging system is proposed by studying the actual UICC system.

**Results:** According to the new staging system, the very heterogenous stage IV from the UICC TNM-classification is now divided in two subgroups, stage IVA and IVB. The stage IVA is restricted to a homogenous group what prognosis concerns including only T3-T4 and N2-N3 M0 patients. The stage IVB includes all patients with any T and a supraclavicular lymph node metatases as well as all patients with an M1 stage. Stage III includes T3-T4 N1 M0 and T2 N2-N3 M0 patients. Stage II includes T3-T4 N0 M0 and T2 N2-N3 M0 patients. Stage I is restricted to T1-T2 N0 M0 patients.

**Conclusions:** This new staging system allows a proper classification of patients with similar prognosis, and will allow adequate selection of patients for treatment, to evaluate results of management more reliable and to compare statistics reported.

### 5.3

**Salivary gland carcinoma: When is the neck at risk?**

H. Lubsen[1], C.H.J. Terhaard[2], [1]Dept. of Otolaryngology, [2]Dept. of Radiotherapy, University of Utrecht, The Netherlands

**Purpose:** In a Dutch multicentric retrospective study (1985-1994) necknode metastases in salivary gland carcinomas were evaluated.

**Material and methods:** Results were obtained of 568 patients with a mean follow-up of 5 years (parotid 59%, submandibular 14%, oral cavity 24%, elsewhere 3%). The distribution according to T-stage was: T1 26%, T2 44%, T3 19% and T4 11%. The histologic type was adenoid cystic carcinoma in 26%, adenocarcinoma NOS 24%, mucoepidermoid carcinoma 16%, acinic cell carcinoma in 12%, carcinoma in pleomorphic adenoma 8%, undifferentiated carcinoma 7%, squamous cell carcinoma 6%.

Clinically positive necknodes were diagnosed in 85 (15%) of all patients. As part of the initial treatment a total of 194 neckdissections was carried out, 63 clinically N+ patients and 131 in clinically N0 patients. According to their N-status all 568 patients were divided into three groups: pN+ (24%), pN0 (15%) and clinically N0 (61%). Regional recurrences in these groups developed in 23%, 10% and 6% respectively.

**Results:** A significant correlation was shown for the presence of positive nodes and T-stage (T1 15%, T2 26%, T3 33%, T4 33%, p=0,00004), site of the primary tumor (oral cavity 9%, parotid 25%, submandibular gland 42%, p<0,00001) and histologic type (acinic cell carcinoma 12%, adenoid cystic carcinoma 15%, carcinoma ex pleomorphic adenoma 22%, mucoepidermoid carcinoma 23%, adenocarcinoma NOS 35%, undifferentiated carcinoma 37% and squamous cell carcinoma 39%, p=0,0002).

**Conclusion:** Patients with high T-stage, tumors in the submandibulair gland, adenocarcinoma NOS, undifferentiated carcinoma and squamous cell carcinoma carry the highest risk of neck node metastases in salivary gland cancer. In these patients treatment of the clinically N0-neck is recommended.

### 5.4

**Prognostic significance of N. XII-resection for submandibular gland adenoid cystic carcinoma (ACC)**

U. Schröder, M. Jungehülsing, H.E. Eckel, E.Stennert, Dept. of ORL, University of Cologne, Germany

**Background:** With respect to the perineural spread and the higher incidence of regional metastases in submandibular gland ACC than that reported for the parotid gland several authors recommended N.XII-resection for treatment of submandibular gland ACC.

**Objective:** To determine if patients without N.XII-resection had a higher incidence of regional recurrences.

**Patients:** We retrospectively reviewed the charts of 254 patients treated for maligne salivary gland neoplasms during 1965-1995 in our clinic according to TNM-stage, N.XII-resection, postoperative radiotherapy and final outcome. 14 out of 24 patients with submandibular gland ACC could be followed up (1-32 years).

**Results:** 5/14 are tumor-free (6-19 years): 2/5 ($T_3N_{0-1}M_0$) with, 3/5 ($T_1N_{0-1}M_0$) without N.XII-resection. Additionally, 1/14 ($T_2N_0M_0$) without N.XII-resection showed no loco-regional recurrence, but pulmonary metastasis after 5 years.

4/14 got loco-regional recurrences: 2/4 ($T_{1-2}N_{0-1}M_0$) are tumor-free for 8-32 years after a single revision-operation sparing the N.XII. 2/4 ($T_{1-3}N_0M_0$) showed recurrence-free intervalls of 4-11 years after revision-operation with N.XII-resection.

4/14 patients ($T_{3-4}N_{0-1}M_0$) died from cancer irrespectively to therapy within 1 year.

Postoperative radiotherapy was given in 7/14 patients and could not be correlated to the incidence of recurrences.

**Conclusion:** In our opinion patients with early stages ($T_{1-2}N_{0-1}M_0$) of submandibular gland ACC may be treated sparing the N.XII.

### 5.5
### Distant metastases in salivary gland carcinoma: The nail in the coffin?

H. Lubsen[1], C.H.J. Terhaard[2], [1]Dept. of Otolaryngology, [2]Dept. of Radiotherapy, University of Utrecht, The Netherlands

**Purpose:** In a Dutch multicentric retrospective study (1985-1994) prognostic factors for distant metastases were studied.
**Material and methods:** 568 patients were analysed (parotid 59%, submandibular 14%, oral cavity 24%, elsewhere 3%). The distribution according to T-stage was: T1 26%, T2 44%, T3 19% and T4 11%. Clinically positive necknodes occurred in 15% of the patients. The mean follow-up was 5 years. For statistical analysis the Cox-PH regression analysis was used.
**Results:** At presentation distant metastases were present in only 17 (3%) patients. Independent factors were T-stages (p=0,001) and clinically positive necknodes (p=0,0001). Actuarial distant metastatic free survival after 8 years was 64%. Independent prognostic factors were: T-stage, (T1-4: 83%, 65%, 59% and 26% respectively), N-stage ($N_0$ 69% and $N_+$ 39%) and histological type (mucoepidermoid carcinoma 85%, acinic cell carcinoma 81%, squamous cell carcinoma 64%, adenoid cystic carcinoma 63%, carcinoma ex pleomorphic adenoma 57%, adenocarcinoma NOS 56% and undifferentiated carcinoma 35%). Survival is longer in patients with distant metastases of adenoid cystic carcinoma as compared to other histologic types: 5 years after the diagnosis of distant metastases 31% versus 0%. The distribution of the distant metastases was as follows: lung 43%, skeleton 20%, liver 6%, brain 1%, elsewhere 6% and multiple of these sites 24%.
**Conclusion:** Although distant metastases in salivary gland cancer are rare at presentation (3%) one out of every three patients will die of distant metastasis eventually.

### 5.6
### Proliferative activity and metastases in adenoid cystic carcinomas of the salivary glands

S. Nordgård, G. Franzén*, M. Boysen**, Depts. of ORL and *Plastic Surgery, University of Trondheim, **Dept. of ORL, The National Hospital, Oslo, Norway

**Purpose:** To study the predictive value of proliferative activity for the early development of metastases in adenoid cystic carcinomas.
**Material and methods:** The proliferative activity was studied by the expression of the Ki-67 antigen in formalin-fixed, paraffin-embedded tissue from 44 patients treated for adenoid cystic carcinoma of the salivary glands. The results were compared with DNA ploidy, clinical factors, histopathological grading and the development of metastases after primary treatment.
**Results:** Metastases developed in eight patients, none in those with free surgical margins at surgery. In the analysis of the disease-free period, high Ki-67 expression was the only significant prognostic parameter (p<0,01). When limited to tumors with violated surgical margins (n=25), high proliferation still predicted early metastases (p=0,01). Free margins were more difficult to obtain in tumors with high proliferative activity even when adjusted for size (T1/T2). Ki-67 expression was significantly related to ploidy and histological grade, but not to tumor size or stage.
**Conclusions:** High proliferative activity in the primary tumor increases the risk for developing early metastases after treatment for adenoid cystic carcinoma of the salivary glands and reduces the chance to obtain free surgical margins at primary surgery. These patients should be followed closely for early detection of metastases irrespective of tumor size and grade.

### 5.7
### Risk factors for metastasis in squamous cell carcinoma of the skin

J. Wehner-Caroli, H. Breuninger, Dept. of Dermatology, University of Tübingen, Germany

Aim of the study was to determine histological and clinical parameters correlating with high risk for metastizing of squamous cell carcinoma (SCC) of the skin.
Course of disease was observed in 822 tumors and compared to clinical and histological features of the underlying SCC.
Following datas were recorded: immunsuppression, localisation, entity, tumor thickness (Breslow), clinical size, depth of invasion (Clark), degree of differentiation (Broders).
Course of disease was evaluated by computerised data based follow up investigations (Tumor Center Tuebingen). The mean lapse of obervation was 4 years. The rate of metastasis over all carcinomas was 5,5% (45 tumors).
Our study shows that the following risk classes can be defined according to rates of metastasis:
-immunsuppression: 32,1% (rate of metastasis) versus no immunsuppression: 4,5%;
-desmoplastic SCC: 23,2% vs. „common" SCC: 4,2%;
-degree of differentiation (Broders) G4: 20,3% vs. G1: 2,2%;
-tumor thickness (Breslow) >5mm: 20,0% vs. 2-5mm: 5,4% and <2mm: 0%;
-clinical size (mean of greatest diameter and the greatest diameter perpendicular on it) >20mm: 12,7% vs. <20mm: 3,3%
-Clark level VI - VIII: 12,3% vs. Clark level I-V: 4,2%
-localisation helix: 10,9% vs. lower lip: 7,2%, remaining head : 4,9% and trunk: 2,3%.
The results show the importance of regular follow up investigations of squamous cell carcinomas of the skin with one or more clinical or histological criterias shown as above.